# Public Beliefs About Accessibility and Quality of Emergency Departments in Germany

**DOI:** 10.5811/westjem.18224

**Published:** 2024-05-03

**Authors:** Jens Klein, Sarah Koens, Martin Scherer, Annette Strauß, Martin Härter, Olaf von dem Knesebeck

**Affiliations:** * University Medical Center Hamburg-Eppendorf, Institute of Medical Sociology, Hamburg, Germany; † University Medical Center Hamburg-Eppendorf, Department of General Practice and Primary Care Hamburg, Germany; ‡ University Medical Center Hamburg-Eppendorf, Department of Medical Psychology, Hamburg, Germany

## Abstract

**Background:**

It is well established that emergency department (ED) crowding leads to worse health outcomes. Although various patient surveys provide information about reasons to visit EDs, less is known in terms of beliefs about EDs among the general population. This study examines public beliefs regarding accessibility and quality of EDs and their associations with social characteristics (gender, age, education, immigration background) as well as knowledge about emergency care services and health literacy.

**Methods:**

We conducted a cross-sectional study based on a random sample of 2,404 adults living in Hamburg, Germany, in winter 2021/2022. We developed eight statements regarding accessibility and quality of EDs leading to two scales (Cronbach’s α accessibility = 0.76 and quality of care = 0.75). Descriptive statistics of the eight items are shown and linear regression were conducted to determine associations of the two scales with social characteristics as well as knowledge about emergency care services and health literacy (HLS-EU-Q6).

**Results:**

Nearly 44% of the respondents agreed that “you can always go to an ED, if you do not get a short-term appointment with a general practitioner or specialist.” And 38% agreed with the statement, “If you do not have the time during normal practice hours due to your work, you can always go to an ED.” In terms of quality, 38% believed that doctors in EDs are more competent than doctors in general practice, and 25% believed that doctors in EDs are more competent than doctors in specialized practices. In the fully adjusted model, public beliefs about emergency care accessibility and quality of EDs were significantly associated with all social characteristics and knowledge of emergency care options with the strongest associations between knowledge and accessibility (β = −0.17; *P* < 0.001) and between education and quality (β = −0.23; *P* < 0.001).

**Conclusion:**

We found endorsement of public beliefs about accessibility and quality of EDs that can lead to inappropriate utilization. Our results also suggest that knowledge of different emergency services plays an important role. Therefore, after system-related reorganizations of emergency care, information campaigns about such services tailored to socially deprived populations may help alleviate the issue of crowding.

Population Health Research Capsule What do we already know about this issue?

*Crowded EDs are associated with poor health outcomes. Patient surveys have shown problematic assumptions about ED accessibility and quality.*

What was the research question?

*We sought to examine beliefs about the ED and their associations with various characteristics in a population survey.*

What was the major finding of the study?

*44% of respondents agreed, “you can always go to an ED, if you can’t get an appointment with an office doctor or specialist,” and 38% said you could use the ED for care during non-business hours.*

How does this improve population health?

*By understanding inappropriate ED use, we can develop education programs for vulnerable groups to inform them about alternative venues to obtain care.*

## INTRODUCTION

Crowding of emergency departments (EDs) has become an important issue in many countries.^1^^–^^3^ Two contributing causes of crowding are boarding of admitted patients (primary) and inappropriate utilization of the ED for non-urgent conditions (secondary).^3^^–^^5^ In terms of the first cause, access block (ie, access to hospital beds is blocked and no admission to an inpatient ward is possible) and hospital admissions for ambulatory care-sensitive conditions (ACSC) have been extensively discussed.^6^^,^^7^ In this study we aimed to address the second cause. Among Organization for Economic Cooperation and Development countries, the increase of ED use in Germany is comparatively high.^8^

Emergency department crowding has been shown to negatively affect patient safety.^3^^–^^5^ Various studies have examined associations between crowded EDs and worse healthcare outcomes (eg, delays in critical treatments, medication errors, return visits, complication rates, and mortality).^9^^–^^11^ For instance, recent research in the United Kingdom found that ED crowding is associated with treatment delay and an increase in all-cause, 30-day mortality.^12^ To reduce patient numbers in EDs, research is focused on avoidable ED visits of patients with non-urgent conditions. Studies have shown that the percentage of all ED visits judged to be non-urgent is about 30–40%, even though study designs were very heterogeneous.^13^ Moreover, a study from Germany found that more than half of the patients visiting an ED did not think that their condition required urgent treatment and thus did not meet the definition of a medical emergency.^14^

In numerous patient surveys, different reasons for visiting EDs with non-urgent conditions were reported. Access barriers to outpatient care, assumptions of higher quality of care and more healthcare options at EDs (as well as negative perceptions about primary care physicians), perceived need and anxiety, convenience (eg, 24/7 availability, no appointments, transport), and referral from healthcare professionals were most frequently mentioned in various international surveys.^13^^,^^15^^–^^17^ Patient surveys conducted in Germany found four main motivations for patients who self-referred to the ED: distress/perceived urgency; access; quality of care; and convenience.^14^^,^^18^^,^^19^

A lower socioeconomic status (SES)—mostly assessed by educational level, income, occupation on individual or regional level, and immigration status—predict more frequent ED utilization and a higher use for low-acuity presentations,^20^^–^^23^ even though some current findings did not completely confirm these inequalities for Germany.^19^ In this context, the concept of health literacy plays an important role.^24^ Low health literacy was shown to be associated with preventable ED visits due to minor or non-urgent problems and with more frequent utilization of EDs and emergency services,^25^^–^^27^ although some other studies did not show this association.^28^

While current evidence provides information about reasons and predictors of frequent or inappropriate ED use, nearly all findings are derived from patient surveys that were conducted at EDs or were based on ED records. These surveys examine the recorded healthcare utilization of actual patients rather than the beliefs about EDs among the general population. This research gap concerning public beliefs about EDs and their accessibility and quality was our rationale for conducting this study. Public beliefs about emergency care are highly relevant as they may contribute to a better understanding of inappropriate ED use and to the development of campaigns to improve health literacy.^29^ Against this background, we explored two research questions: 1) What are the public’s beliefs about EDs in terms of accessibility or convenience and quality of care; and 2) Are there variations in these beliefs about EDs according to social characteristics (age, gender, education level, and immigration status) and health literacy (general health literacy and knowledge of emergency care options)?

## METHODS

### Study Design and Population

A cross-sectional telephone survey was conducted in Hamburg, Germany in winter 2021/2022 via computer-assisted telephone interviews. We obtained a random sample of German-speaking people aged ≥18 years using all possible telephone numbers in Hamburg, including non-registered numbers, via random digital dialing. Only landline numbers could be included as mobile telephone numbers are not provided on a regional level. About 83% of all households in Germany have a landline telephone.^30^ Thus, a large majority of the population can be reached via landline numbers. Repeated calls were made by trained interviewers of a professional survey research institute (USUMA, Berlin, Germany) on different weekdays. We applied the Kish selection grid to randomly select the target person in the contacted households.^31^ Prior to this, the same survey research institute conducted a pilot study among 30 individuals in the general population.

We chose a telephone survey as the method for data collection due to the vignette design of the study. At the beginning of the survey, recorded audio files describing different symptoms were directly played to the respondents. To guarantee a standardized stimulus and immediate response, telephone surveys are usually favored and an established method.^32^ Subsequently, a standardized questionnaire was applied. Sample size was calculated based on a vignette design (48 vignettes in total) applied in the study. According to power calculations, a sample size of 50 respondents per vignette was calculated to identify medium size differences resulting in about 2,400 required participants (statistical power 0.8, and type-I error 0.05). These vignettes were not used in the present analyses. The sample consisted of 2,404 respondents.

Due to different approaches for the definition of eligibility in telephone surveys, different response rates (RR) can be calculated.^33^ Thus, a RR in this survey varied between 10.9–46.0% (American Association of Public Opinion Research RR3^34^ 17.3%). To gain a representative sample, we weighted data for household size, gender, age, educational level, and place of residence (district in Hamburg) using the official statistics regarding the adult population living in Hamburg.^35^^–^^37^ In accordance with Halbesleben and Whitman,^38^ we conducted a sample/population comparison to assess nonresponse bias. Table 1 shows that the weighted sample adequately represents the general adult population of Hamburg regarding the distribution of gender, age, and educational level.^35^^,^^36^ The survey was approved by the Local Psychological Ethics Committee at the Center for Psychosocial Medicine, University Medical Center Hamburg (No. LPEK-0200). Respondents gave their informed consent for the participation and the use of their data. Consents and refusals were documented by the interviewers.

**Table 1.** Sample characteristics of survey respondents compared with official statistics for the population in Hamburg by percentage.

	Sample^a^ (N = 2,404)	Adult population of Hamburg 2020^b^	*P* ^c^
Gender *(0)* ^d^			
Male	48.5	48.4	0.95
Female	51.5	51.6	
Age (years) *(0)*			
18–24	9.6	9.4	0.83
25–34	19.7	19.6	
35–44	17.2	17.5	
45–54	17.5	16.6	
55–64	14.1	15.1	
65–74	10.1	10.0	
≥75	11.8	11.8	
Education level *(71)*			
low	27.4	27.0^e^	0.32
middle	24.1	24.1	
high	48.5	48.9	
Migration background *(46)*			
No migration background	77.9	66.8	–^f^
2^nd^ generation	11.2	–^g^	
1^st^ generation	10.9	–^g^	

^a^ Weighted;

^b^ 34,35;

^c^ Pearson’s chi^2;^

^d^ Number of missing cases in brackets in italics;

^e^ Data for education only available for people ≥15 years old.

^f^ No exact data available.

^g^ As there was no discrete weighting for immigration background, test statistics were not conducted.

### Measures

**Figure 1.**
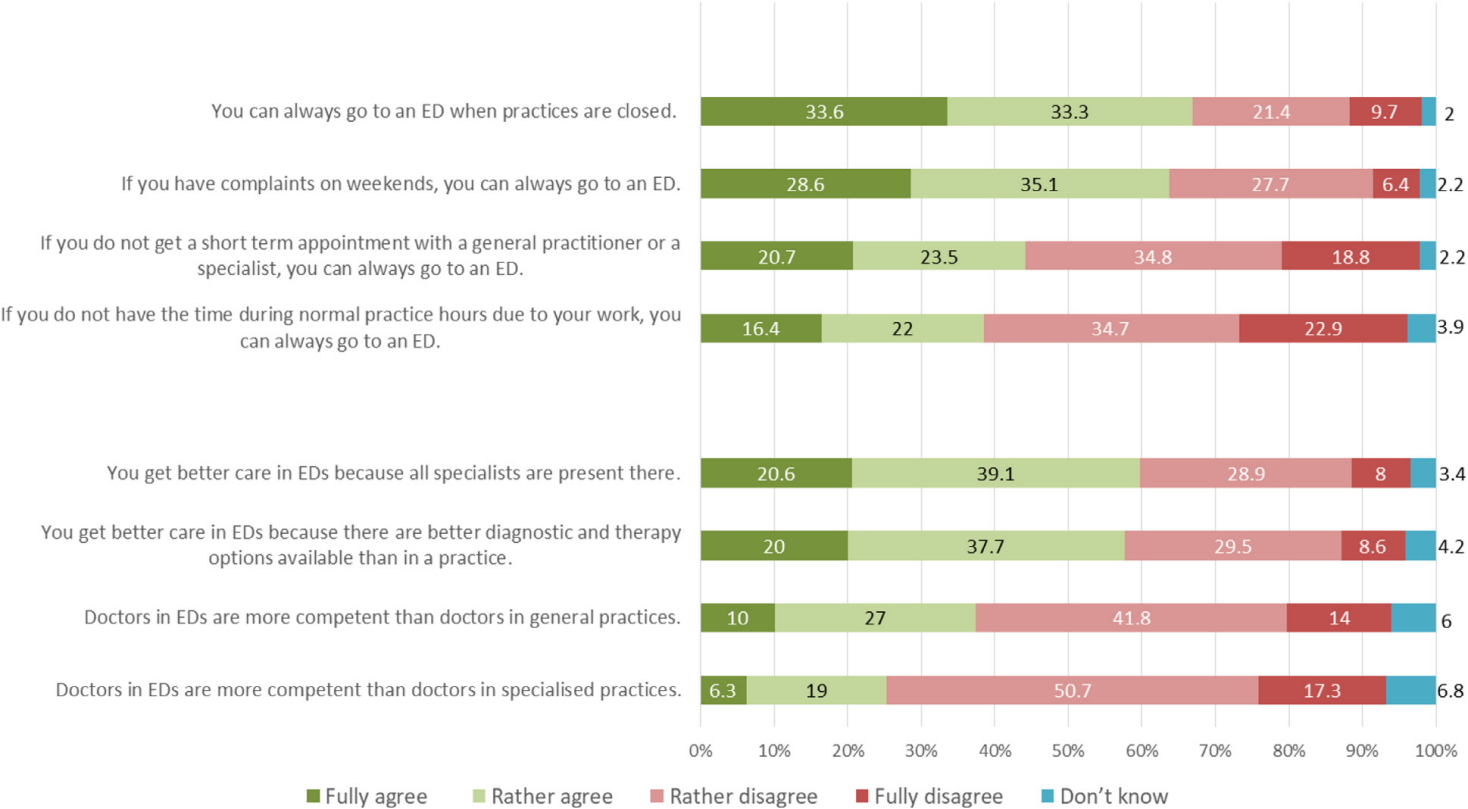
Public beliefs about accessibility and quality of emergency departments (N = 2,404). *ED, *emergency department.

To assess the public’s beliefs about EDs, we developed eight items (statements about EDs) based on a review of the literature.^13^^–^^16^^,^^18^^,^^19^^,^^39^ As described above, the main motivations for preferring EDs in patient surveys were related to barriers to access of outpatient care, convenience, assumptions of higher quality of care, and distress or subjective need. We developed four statements regarding access barriers and convenience, as well as four statements related to the quality of care provided in EDs (Figure 1). As the survey was conducted among the general population and not acute patients in EDs, we did not include statements regarding distress and subjective need. Response categories were “fully agree,” “rather agree,” “rather disagree,” “fully disagree” and, additionally, “don’t know,” with higher values indicating stronger agreement. Validity was tested in accordance with some aspects of Messick's unified framework.^40^

We collected content validity evidence through an extensive literature screening of patient surveys identifying the main motivations for preferring EDs. Additionally, experts in emergency care were involved in the item development. Using a pilot study, we pretested items and response consistencies. Furthermore, internal structure validity was tested through Cronbach’s α and the factorial structure of the instrument via principal component analysis. The weighting of demographics in our study was aimed to meet external validity.

Gender, age, educational level, and immigration background (no immigration background, first generation, second generation) were introduced as social characteristics of the respondents. Education level was based on years of schooling (9 years = low; 10 years = middle; ≥12 = high). A person was considered to have an immigration background if he or she or at least one parent was born abroad. Respondents with an immigration background who were born in Germany are considered second-generation immigrants, while those with immigration experience are subsequently termed first-generation immigrants. We assessed general health literacy using a European health literacy survey questionnaire, the HLS-EU-Q6, a short form of the established HLS-EU-Q47.^41^ On a four-point Likert scale, the answer categories were “very difficult,” “fairly difficult,” “fairly easy,” and “very easy,” including “don’t know.” The Cronbach’s α = 0.60 of the scale is acceptable for an instrument that is short and features discrete elements of literacy.^42^ We computed a sum scale by averaging the responses to the six items resulting in a range between 1–4, with higher scores indicating an increased health literacy.

To specifically assess knowledge about available emergency care services, we asked the respondents to name all options of emergency care they knew of (open-ended question). In the German healthcare system, patients basically have four options of emergency care.^43^ They can 1) call the rescue service (telephone number 112); 2) go to an ED; 3) go to an emergency practice (practices that are usually open from 6 pm to midnight for urgent conditions); or 4) contact the medical on-call service (also known as “116 117,” referring to the telephone number) in urgent or emergency cases. In the survey, this question was located before the item about a respondent’s beliefs concerning EDs. Based on the responses (respective emergency care service mentioned = 1, not mentioned = 0), we calculated a sum scale with a possible range from 0–4 with higher scores indicating more knowledge.

### Analyses

We present percentages of agreement of the eight single items to assess beliefs about EDs as descriptive results. Furthermore, we conducted a principal component analysis including the eight items assessing public beliefs. The analysis revealed two components with eigenvalues ≥1 reflecting accessibility (eigenvalue: 3.22, explained variance: 40.3%) and quality of care (eigenvalue: 1.41, explained variance: 17.6%), which accounted for 57.9% of the total variance (rotated loadings between 0.66–0.78). Eigenvalues are used to determine the relative importance and the explained variance of each principal component. Usually, only factors with eigenvalues ≥1 are considered.^44^ Accordingly, for the multivariate analyses, two scales representing access barriers and convenience (subsequently labelled as “accessibility,” four items) and quality of care (“quality,” four items) were calculated ranging from 1–4. Higher scores indicate stronger agreement with easy accessibility and superior care quality with regard to EDs. In terms of reliability, internal consistency of the two scales revealed satisfactory results (Cronbach’s α = 0.76 [accessibility] and 0.75 [quality]).

We calculated linear regression models to analyze associations between social characteristics, health literacy, and public beliefs about accessibility and quality of EDs. Dependent variables were the two scales regarding accessibility and quality of EDs. As predictor variables, gender, age, education level, immigration background, general health literacy, and knowledge of emergency services were introduced. In a first step, we calculated simple unadjusted models showing the single estimates and significances of each predictor variable. Thereafter, in the full model, the predictor variables were entered simultaneously adjusting all variables for each other. We documented regression estimates (B), standardized B (β), significances (*P*), and explained variance (R^2^). Results with *P* < 0.05 were considered statistically significant. As many participants chose the option “don’t know” when completing the HLS-EU-Q6, which had to be considered as missing value, the multivariate analyses were conducted with a sample size of 1,751 (quality) or 1,826 (accessibility), respectively. Moreover, various key assumptions for linear regression models (linear relationship, normal distribution of residuals, auto-correlation, homoscedasticity, and multicollinearity) were successfully tested. All analyses were calculated with weighted data and carried out using the Statistical Package for the Social Sciences V 27 (SPSS, Inc, Chicago IL).^45^

## RESULTS

Mean age of the respondents was 48.8 years (SD 19.0); 51.5% were female. Almost half of the sample (48.5%) had a high educational level (middle level: 24.1%; low level: 27.4%). About 11% each belonged to the group of first- or second-generation immigrants, while about 78% of the sample had no immigration background (Table 1). The mean (SD) was 2.56 (0.49) for health literacy (HLS-EU-Q6) score (range 1–4). Regarding knowledge of available emergency services, on average the respondents knew two of four options. Figure 1 shows the distribution of the eight items measuring beliefs about accessibility and quality of EDs. Agreement (percentage of respondents who “fully” and “rather” agreed summed up) to the items related to an easy access of EDs ranged between 38.4% (“If you do not have the time during normal practice hours due to your work, you can always go to an ED”) and 66.9% (“You can always go to an ED when practices are closed”). In terms of quality of care provided in EDs, 25.3% of the respondents “fully” or “rather” agreed with the item “Doctors in EDs are more competent than doctors in specialized practices,” while 68% “rather” or “fully” disagreed. Regarding the item “You get better care in EDs because all specialists are present there,” 59.7% expressed agreement.

Table 2 shows the results of linear regression analyses with the sum scale indicating accessibility of EDs as the dependent variable. As can be seen in the unadjusted models, all predictor variables indicated significant associations with beliefs about accessibility. Strongest associations were shown for education level and knowledge of emergency care service. In the fully adjusted model, female respondents less often agreed that EDs are characterized by easy accessibility. Moreover, agreement increased with age, while it decreased with education level and knowledge of emergency care service options. Compared to respondents without an immigration background, first- and second-generation immigrants more strongly believed in the easy accessibility of EDs. Highest β-values in the fully adjusted model were shown for education (β = −0.13, *P* < 0.001), knowledge (β = −0.17, <0.001) and age (β = 0.13, *P* < 0.001).

**Table 2.** Beliefs about emergency departments: sum scale accessibility^a^ (N = 1,826^b^) (linear regressions).

	Unadjusted models			Fully adjusted model		
Predictor variables^c^	B	β	p	B	β	P
Gender	−0.178	−0.12	<0.001	−0.156	−0.10	<0.001
Age	0.006	0.16	<0.001	0.005	0.13	<0.001
Education	−0.197	−0.22	<0.001	−0.116	−0.13	<0.001
Migration background						
1^st^ generation	0.372	0.15	<0.001	0.275	0.11	<0.001
2^nd^ generation	0.135	0.06	0.01	0.179	0.08	0.001
Health literacy (HLS-EU-Q6)	−0.90	−0.06	0.01	−0.049	−0.03	0.15
Knowledge of emergency care services^d^	−0.186	−0.25	<0.001	−0.125	−0.17	<0.001
R^2^ (fully adjusted model)					0.122	

B = regression estimate, β = standardized B, *P* = significance (significant associations [*P* < 0.05] are bold).

^a^ Higher values indicate stronger agreement (range 1 to 4).

^b^ All analyses based on the sample size of the fully adjusted model.

^c^ Gender = reference: male, age = range 18–96 years, education = range 1–3; migration background = reference: no migration background; health literacy = range 1–4; knowledge of emergency care services = range 0–4.

^d^ Emergency department/emergency practice/rescue service/medical on-call service.

In terms of beliefs about superior care quality in EDs, significant associations were shown for all predictors except immigration background (second generation) in the unadjusted models (Table 3). Again, education level and knowledge of emergency care service indicated highest β-values. Regarding the fully adjusted model, significant negative associations with education level, emergency care knowledge, and health literacy emerged. Furthermore, these beliefs increased with age and were more pronounced among first-generation immigrants and males. Education level showed the strongest association (β = −0.23, *P* < 0.001) in the fully adjusted model.

**Table 3.** Beliefs about emergency departments: sum scale quality^a^ (N = 1,751^b^) (linear regressions)

	Unadjusted models			Fully adjusted model		
Predictor variables^c^	B	β	p	B	β	P
Gender	−0.162	−0.12	<0.001	−0.130	−0.10	<0.001
Age	0.007	**0.19**	<0.001	0.005	**0.13**	<0.001
Education	−0.246	−0.32	<0.001	−0.183	−0.23	<0.001
Migration background						
1^st^ generation	**0.324**	**0.15**	<0.001	0.232	**0.11**	<0.001
2^nd^ generation	0.005	0.05	**0.91**	0.062	0.03	**0.18**
Health literacy (HLS-EU-Q6)	−0.129	−0.10	<0.001	−0.076	−0.06	0.01
Knowledge of emergency care services^d^	−0.144	−0.22	<0.001	−0.073	−0.11	<0.001
R^2^ (fully adjusted model)					**0.155**	

B = regression estimate, β = standardized B, p = significance (significant associations [*P* < 0.05] are bold).

^a^ Higher values indicate stronger agreement (range 1 to 4).

^b^ All analyses based on the sample size of the fully adjusted model.

^c^ Gender = reference: male, age = range 18–96 years, education = range 1–3; migration background = reference: no migration background; health literacy = range 1–4; knowledge of emergency care services = range 0–4.

^d^ Emergency department/emergency practice/rescue service/medical on-call service.

## DISCUSSION

Based on a population survey in a German metropolis, we assessed the public’s beliefs about accessibility and quality of care of EDs. Nearly 44% of the respondents agreed that “you can always go to an ED, if you do not get a short-term appointment with a general practitioner or a specialist.” Still, 38% agreed to the statement “If you do not have the time during normal practice hours due to your work, you can always go to an ED.” In terms of superior quality, 38% believed that doctors in EDs are more competent than doctors in general practice and 25% regarded doctors to be more competent in specialized practices. In addition, nearly 60% agreed that “you get better care in EDs because all specialists are present there.” Furthermore, the public’s perceptions of emergency care are significantly associated with social characteristics (gender, age, education level, immigration background) and knowledge of emergency care options. Regarding accessibility, knowledge showed the strongest association: The more options of emergency care respondents named, the less respondents agreed that EDs are always accessible. In terms of beliefs about quality of care, education level turned out to be the strongest predictor: The less educated the respondents were the more they agreed that the quality of care is superior in EDs.

As there are many patient surveys but very few population-based studies, comparability of our results with previous research is limited. Some researchers also aimed to assess public perceptions about EDs, but their methods vary considerably.^27^^,^^46^ Regarding attitudes toward accessibility and quality, males, older people, ethnic minorities, and people with lower SES showed a tendency to use emergency services, even for minor problems, more frequently. An Australian study among the general population showed that perceived urgency, good accessibility, and better healthcare provision were stated as reasons to visit an ED.^46^ However, no further analysis about predicting factors was conducted. In a British survey using case vignettes, the tendency to call for an ambulance or to visit an ED in less urgent cases was significantly increased for males, older age, and those who were of ethnic minorities and had a lower paid occupation and a lower level of health literacy.^27^

When developing the statements concerning accessibility, we deliberately chose strict wording (“always”), so that the items were not too leading. To agree to the four statements was not completely wrong, but it was inappropriate in terms of favored navigation within the German healthcare system. Services in the ED are generally provided for life-threatening conditions or serious acute problems that cannot wait and need to be treated by a doctor immediately. In less urgent cases, other alternatives should be preferred. For these cases, mainly two services are provided when practices are closed: emergency practices and the medical on-call service (also known as “116 117” referring to the telephone number). In fact, these two services were implemented to unburden EDs. This was taken into account when we developed the four statements concerning beliefs about accessibility. It is similar in the case of the statements regarding better quality of care in EDs. There is concentrated expertise in hospitals, but the rating of worse expertise of outpatient doctors and the assumption that all specialists are available in the emergency ward are doubtful and could lead to unrealistic expectations regarding the use of EDs. In this regard, the present study could help us to understand the public’s beliefs on which inappropriate utilization of EDs are based. However, it cannot be ruled out that some participants did not correctly understand the items.

The findings provide data about the lack of health education among the general population. Particularly, males, older and less educated people, and those with limited knowledge of emergency care options showed a potentially inappropriate utilization of the ED. In terms of immigration status, especially first-generation, immigrants showed a lack of information that could be due to less experience with the healthcare system, language barriers, different expectations and preferences, as well as formal access barriers (eg, waiting times or travel distances).^47^ Regarding gender-specific differences, previous research showed a higher ED attendance for non-urgent problems and a higher use of out-of-hours help-seeking among men.^27^^,^^48^ Potentially, this preference could be due to longer working hours among men and less willingness to be absent from work because of healthcare. Thus, social inequalities should be considered when implementing interventions (eg, information campaigns). To modify public beliefs about healthcare in general or emergency care in particular, “emergency literacy” campaigns are a way to address the problem of ED crowding. In this regard, knowledge about the availability of different emergency care services, navigation within the healthcare system, and the assessment of symptoms could be addressed.

People should be educated that the ED is for life-threatening and serious conditions such as heavy bleeding, broken bones, chest pain or stroke, and that many symptoms can be treated more appropriately elsewhere. An Australian behavior change campaign that focused on attitudes, awareness, and knowledge was successful in reducing the number of inappropriate or non-urgent calls to ambulance services or medical emergency phone numbers.^29^ Currently, a qualitative study from Germany positively evaluated an educational intervention tailored for ED patients with low-acuity conditions.^49^ Another study examined physician-directed strategies for improving patient health literacy in EDs.^50^ Furthermore, lower health literacy was found among people who were of older age and had lower education levels, less affluence, and with immigration backgrounds,^51^^,^^52^ which are factors that were also shown to be associated with higher and inappropriate ED use in some studies.^25^^–^^27^ As our data of public beliefs supports the findings of social inequalities in inappropriate ED use, tailored health education has to take place in more deprived areas where vulnerable groups are living and the availability of healthcare services is potentially limited. Information in different languages and in digital and non-digital versions could help to reach the population in a better way.

In this study, we focused on beliefs that may foster an inappropriate utilization of the ED for non-urgent conditions as one cause of crowding. Another and more important reason is related to boarding of admitted patients.^3^ In this context, access block and hospital admissions for ambulatory care-sensitive conditions (ACSC) are discussed.^6^^,^^7^ Access block is the situation in which access to hospital beds is blocked and no admission to an inpatient ward is possible.^6^ Hospital admissions for ACSC are defined as admissions in hospital wards including EDs for medical conditions that are potentially avoidable if they are managed in the outpatient care.^7^ Through ACSC, the availability, access and quality of outpatient care can be evaluated, and social inequalities can be revealed.^7^ Some reviews summed up possible implications and interventions in terms of reorganization of ED wards and availability of outpatient care.^53^^–^^55^ Recently, reforms of emergency care have been discussed in Germany in terms of allocating and triaging patients (ie, implementation of a coordination center for first telephone contact and further allocation, and a general counter for initial assessment and triaging at EDs).

## LIMITATIONS

This study has some limitations that need to be discussed. Even though the data was weighted for gender, age, and education level, and the comparison of sample and population showed reasonable results, a potential selection bias due to non-response and the exclusive use of landline numbers cannot be ruled out. In this regard, a response rate of between 10.9–46% (depending on definition of eligibility) can be considered acceptable compared to other telephone surveys.^56^ Moreover, 83% of households can be reached via landline numbers in Germany.^30^ Our data refers to the situation of healthcare provision in a German metropolis. The conditions in other countries and in more rural regions could be different.

As there was no validated measure for public beliefs about availability of EDs, we developed eight items based on a review of the literature of patient surveys. Although psychometric properties of the two scales seem adequate (Cronbach’s α = 0.76 [accessibility] and 0.75 [quality]),^42^ these scales need to be further developed and tested. In terms of missing values, some items of the accessibility and quality scale (n = 185 and n = 320), and notably items of the HLS-EU-Q6 scale yielded a high number of missing values due to “don’t know” answers (434). Although this procedure was in accordance with the original HLS-EU instrument, the option of “don’t know” in questionnaires should not be automatically treated as missing values. Therefore, a missing analysis was conducted. The results revealed only a consistent pattern for age (significantly increased missing values among people with older age). Thus, the relevance of age could be underestimated in the regression analyses, and due to subjective data a common method bias could not be ruled out. Finally, the evaluation of general health literacy was conducted with an established instrument of the HLS-EU consortium, but with the shortest version available (HLS-EU-Q6).^41^ So, a more comprehensive instrument would possibly lead to an improved assessment.

## CONCLUSION

This study suggests that the public’s perceptions about ED quality and accessibility contribute to inappropriate ED utilization and crowding in Germany. Particularly, this holds true for people of older age, male gender, lower education level, and those who are first-generation immigrants and who have less knowledge about available emergency care services. The findings help in understanding inappropriate utilization of emergency care services and developing health education programs tailored to socially deprived populations.
